# Depression and anxiety in peruvian military personnel during the pandemic context: a cross-sectional study

**DOI:** 10.1186/s12889-023-15612-z

**Published:** 2023-04-13

**Authors:** Mario J. Valladares-Garrido, Cinthia Karina Picón-Reátegui, J. Pierre Zila-Velasque, Pamela Grados-Espinoza, Víctor J. Vera-Ponce, César Johan Pereira-Victorio, Danai Valladares-Garrido, Virgilio E. Failoc-Rojas

**Affiliations:** 1 Escuela de Medicina, Universidad Cesar Vallejo, Piura, Peru; 2 Oficina de Epidemiología, Hospital Regional Lambayeque, Chiclayo, Peru; 3 Faculty of Medicine, Universidad de San Martín de Porres. Chiclayo, Santa Anita, Peru; 4 Facultad de Medicina Humana, Universidad Nacional Daniel Alcides Carrión, Pasco, Peru; 5 Red Latinoamericana de Medicina en la Altitud e Investigación (REDLAMAI), Pasco, Peru; 6 Instituto de Investigación en Ciencias Biomédicas, Universidad Ricardo Palma, Lima, 15039 Peru; 7 Universidad Tecnológica del Perú, Lima, 15046 Peru; 8 School of Medicine, Universidad Continental, Lima, Peru; 9 Unidad de Epidemiología y Salud Ambiental, Hospital de Apoyo II Santa Rosa, Piura, Peru; 10 Research Unit for Generation and Synthesis Evidence in Health, Universidad San Ignacio de Loyola, Lima, Peru

**Keywords:** COVID-19, Mental Health, Military, Latin Americans, cross-sectional

## Abstract

**Background:**

During the COVID-19 pandemic, increased workload and stress could have increased mental health problems (anxiety and depression) in military personnel. However, the number of studies in military members is scarce, especially in regard to mental health. The objective of this study was determine the prevalence and factors associated with depression and anxiety in Peruvian military personnel.

**Methods:**

We undertook an analytical cross-sectional study. The survey was distributed face to face between November 02 and 09, 2021, during the second wave of the COVID-19 pandemic among the military personnel. We used some instruments to measure depression (Patient Health Questionnaire, PHQ-9), anxiety (Generalized Anxiety Disorder, GAD-7), insomnia (Insomnia Severity Index, ISI), food insecurity (Household Food Insecurity Access Scale, HFIAS), physical activity (International Physical Activity Questionnaires, IPAQ-S), resilience (abbreviated CD-RISC), and fear of COVID-19 scale. The exclusion criteria included those who did not completely fill out the evaluation instruments.

**Results:**

We analyzed the data of 615 military personnel that participated in the survey. Of them, 93.7% were male and the median age was 22 years old. There was a prevalence of 29.9% and 22.0% in regard to depression and anxiety symptoms, respectively. In addition, it was found that being married (PR: 0.63; 95% IC: 0.42–0.94), having a relative with mental health problems (PR: 2.16), having experienced food insecurity (PR: 1.48), insomnia (PR: 2.71), fear of COVID-19 (PR: 1.48), and a high level of resilience (PR: 0.65) were factors associated with depression. In regard to anxiety, the factors associated were working for more than 18 months since the beginning of the COVID-19 pandemic (PR: 0.52), a high level of resilience (PR: 0.50; 95% IC: 0.33–0.77), insomnia (PR: 3.32), fear of COVID-19 (PR: 2.43).

**Conclusion:**

We found a prevalence of symptoms of depression and anxiety of 29.9% and 22.0%, respectively. In regard to the factors that attenuate depression, we can mention being married and having resilience; and among the aggravating factors, having a relative with mental health problems, food insecurity, insomnia, and fear of COVID-19. Finally, anxiety increased through working time, insomnia, and fear of COVID-19.

## Introduction

The COVID-19 pandemic has had a negative impact on mental health in general population [1,2] due to radical changes in life conditions and work caused by the isolation measures and mandatory social distancing [3]. The most important psychological effects of the pandemic were short-time anxiety and depression symptoms [4]. During the COVID-19 pandemic, the prevalence of depressive symptoms and anxiety symptoms reported in China, Spain, Italy, Iran, USA, Turkey, Nepal, and Denmark [5] were between 14.6% and 50.9%. In Peru, the prevalence of overall depressive symptoms and anxiety was 16.0% and 11.7%, respectively [6]. In relation to factors associated with depression and anxiety symptoms, diverse studies such as systematic reviews have reported the following: being a female, young, single, divorced or widowed, a low educational level, history of mental health, having an acquaintance infected with COVID-19 [5, 7], lack of confidence related with test security, having had direct or indirect contact with a confirmed case of COVID-19, and having taken prevention measures to avoid COVID-19 spread [7].

During containment of the COVID-19 pandemic, after that Peru declared the State of National Emergency, military personnel have supported the compliance of the prevention measures such as accompanying health personnel in the identification of positive cases and in the care of vaccination centers and hospitals [8]. Military personnel usually carry out high-risk operations in response to emergencies and disasters [9]; and in this health emergency, they played an important role in the first-line of defense, contributing towards the reduction of contagion and deaths due to COVID-19[9]. According to studies, it is known that military personnel are in risk of having mental health problems such as depression and anxiety [10]. A systematic review reported higher levels of anxiety and depression symptoms in military personnel before the pandemic [11]. Another systematic review reported a prevalence of depression of 23.0% among active military personnel [12]. During the COVID-19 pandemic there was a workload and stress increase that could have increased negative mental health symptomatology (anxiety and depression) in military personnel [10]. In military members, according to the USA reports, the prevalence of depressive symptoms and anxiety during the pandemic was 17.4% and 16.8% [13]; in Canada, the prevalence reported during the first months of the pandemic was 14.3% and 14.5% for depression and anxiety, respectively, among active soldiers [14]. In the United Kingdom, during the pandemic, it was reported that the prevalence of mild depression was 30.2%, and of mild anxiety was 21.8% [15]. In the case of Greece, during the first year of the pandemic, it was reported that the prevalence of mild depressive symptoms was 1.3% [16].

In regard to the effects of mental health during the COVID-19 pandemic in military personnel, no studies have been conducted in Latin America. In the USA, different studies in military population were carried out, in which some limitations were identified such as the collection of data in a virtual format [13], the fact that the population corresponded to veterans (military in an inactive status and/or older adults) [17], and a very small sample in an Irish study [18]. In addition, we have not found a study with Latin American data of military population in the first line of defense against COVID, given that most of the evidence documented has focused on first-line medical care [19–21]. Moreover, there does not exist conclusive evidence about the potential factors that influence the symptoms of depression and anxiety such as being a male, older adult, military rank, having any disease [12], excessive alcohol use [11], and the use of masks during the COVID-19 pandemic [13].

Therefore, our study aimed to determine the prevalence and factors associated with depression and anxiety symptoms in military personnel in Lambayeque, Peru.

## Methods

### Study design

We undertook an analytical cross-sectional design and used the data of a survey responded by military personnel of the first line of defense against COVID-19 of the region Lambayeque, Peru. The survey was distributed on site, between November 02 and 09, 2021, in order to evaluate mental health during the context of the second wave of the COVID-19 pandemic.

### Population and sample

The study population included 820 military members who worked actively during the health emergency due to COVID-19 in the region of Lambayeque, Peru. Lambayeque has shown a high seroprevalence [22] and mortality levels [23] due to coronavirus.

We estimated a sample of 582 military personnel, after using a 2.5% precision, 99% confidence level, 12.8% expected prevalence, and 20% for potential missing data or refusals. When we conducted the study, it was feasible to capture the participation of 86.6% (n = 710) of the population, a greater number than we had expected. A snow-ball and convenience sampling was used to recruit the participants.

We included military personnel that, at the moment of requesting participation in the research, were working during the health emergency. Military members that did not give their consent to participate in the study, who were working remotely for having a high risk of COVID-19, and those who were in quarantine for being infected with coronavirus were excluded. Also, 95 military personnel were excluded for not having completely filled out the PHQ-9 instrument (depression) and GAD-7 (anxiety).

### Variables

#### Dependent variables

Depression, defined operatively as a score higher than four points, obtained through the sum of the responses of military personnel participating in the PHQ-9 questionnaire.

Anxiety, defined operatively as a score higher than four points obtained through the sum of the responses of military personnel participating in the GAD-7 questionnaire.

#### Independent variables

Regarding sociodemographic characteristics, we obtained data on age in years, gender (female and male), marital status (single, married, cohabiting, divorced), religion (none, Catholic, non-Catholic), children (no, yes), frequent alcohol consumption (no, yes), previous diseases (arterial hypertension, diabetes), body mass index (underweight, overweight, type 1, 2, 3 obesity).

In regard to psychological characteristics, we obtained the following information: personal and family history of health problem (no, yes), report of having sought mental health support due to the COVID-19 pandemic (no, yes), report of having confidence in the government to manage COVID-19 (no, yes), working time since the beginning of the pandemic (1 to 6 months, 7 to 12 months, 13 to 18 months), food insecurity, measured through the question Over the last four weeks, were you worried about not having enough food at home?

### Instruments

The instruments had seven sections that covered (1) sociodemographic characteristics; (2) psychosocial characteristics; (3) depression questionnaire (PHQ-9); anxiety questionnaire (GAD-7); 5) insomnia questionnaire (ISI); 6) food insecurity questionnaire (HFIAS); 7) questionnaire of physical activity (I-PAQS); 8) Connor Davidson abbreviated questionnaire (CD-RISC); 9) Fear of COVID-19 scale.

#### Depressive symptom questionnaire (PHQ-9)

This questionnaire is a self-administered scale, comprised of nine items, which are directly related with depressive symptoms during two weeks prior to the administration of the scale. Each item is scored according to a Likert scale that goes from 0 (never) to 3 (almost every day). The scores of the PHQ-9 reflect five categories of the severity of the depressive disorder: none (0–4 points), mild (5–9 points), moderate (10–14 points), moderately severe (15–19 points), and severe (20–27 points) [24]. Regarding its psychometric properties, it has 88% sensitivity and specificity when the PHQ-9 ≥ 10; in addition, it has an adequate internal consistency (Cronbach’s alpha = 89) [24]. This questionnaire was validated in Latin America such as Peru; and it was used during the pandemic in diverse studies [25] and in different populations such as interns of human medicine [26].

#### Anxiety symptom questionnaire (GAD-7)

The questionnaire that measures the presence of generalized anxiety disorder is constituted by seven questions about anxiety symptoms in the last two weeks, where each one has a score from 0 to 3 points using the Likert scale. It has a cut-off point of 10 points. By following this criterion, we can identify four categories of anxiety severity: none (0–4 points), mild (5–9 points), moderate (10–14 points), and severe (15–21 points) [27]. It was validated in Latin America and has obtained an optimal internal consistency ( Cronbach’s alpha = 0.92). This instrument has been used in Peruvian health personnel to explore anxiety during the COVID-19 pandemic [19].

#### Insomnia questionnaire (ISI)

It consists of seven items that measure the perception of severity of insomnia through the Likert scale from 0 to 4 points (for example, 0 = none; 4 = very severe), and the final score varies between 0 and 28 points [28]. The total score is interpreted as follows: absence of clinical insomnia (0–7); subclinical insomnia (8–14); moderate clinical insomnia (15–21), and severe clinical insomnia (22–28) [28]. It has been validated in older adults [29] and Spanish-speaking general population [30].

#### Questionnaire of food insecurity (HFIAS)

It was developed by the Agency for International Development of the United States. The scale includes nine items that correspond to questions about food in the last four weeks. In the instructions of the instrument, respondents are asked if they have experienced food insecurity in their household in a determined period, along with the anxiety they may have, the quality of food and insufficient intake as well as the physical consequences. The answers about food insecurity are classified as follows: food security (question 1), mild food insecurity (questions 2 to 4), moderate food insecurity (questions 5 or 6), and severe food insecurity (questions 6 to 9) [31]. This questionnaire has been adapted to Spanish by Coates in 2007, using the US Household Food Security Survey Module (HFSSM) [32]. Likewise, it presents optimal internal consistency, corroborated by Teh et al. (α = 0.88) [33], Naja et al. (α = 0.91) [34], and Knueppel et al. (α = 0.83–0.90) [35]. The Spanish version of the instrument has been validated in the Peruvian population [36].

#### Physical activity questionnaire (IPAQ-S)

It has nine items and evaluates physical activity reported in the last seven days. This instrument allows a weighted estimate of the total physical activity from the activities notified by week. It evaluates three specific characteristics: intensity (mild, moderate, or vigorous), frequency (measured in days per week), and duration (hours a day) [37]. Physical activity was categorized into low or inactive, moderate, and high [38]. It has been validated in Spanish-speaking populations and administered to Latin American population [39].

#### Connor Davidson abbreviated questionnaire (abbreviated version of CD-RISC)

It was used to evaluate resilience. It has ten items and can be used as a reliable and valid instrument. The original version has good properties: Cronbach’s alpha of 0.89 (general population) and a test-retest reliability coefficient of 0.87 (people with generalized anxiety disorder (GAD) and post-traumatic stress disorder (PTSD)) [40]. Resilience was evaluated through a Likert scale with five options with a score which ranges from zero to four. The higher the score, the higher the resilience. In general, it shows excellent psychological properties and permits an efficient measure of resilience [41]. The present instrument was validated in Peruvian population [42].

#### Fear of COVID-19 scale

It has seven items and is reliable and valid to assess fear of COVID-19 among general population, with a Cronbach’s alpha coefficient of 0.82 [43]. It uses a Likert scale from 1 to 5 points, which goes from 1 (strongly disagree) to 5 (strongly agree) and the total scores range from 7 to 35 [44]. A study on the psychometric properties of the Spanish version of Fear of COVID-19 in a sample of Peruvian population demonstrated that this short scale of fear of COVID-19 has an internal consistency coefficient of 0.87, which shows adequate properties of measurement both in reliability and validity [44].

### Procedures

A questionnaire was designed in the REDCap data entry system in order to guarantee optimal quality of registered data. After obtaining the authorization of the commandant of the military site located in Lambayeque-Peru, a field team was constituted in order to go to the military site and administer the instruments. We obtained permission to attend the site in two shifts (morning and afternoon), where all the population gathered in three groups during November 02–09, 2021. Thoroughly, we requested the compliance of the prevention measures against COVID-19, especially the correct use of masks, social distancing, and hand washing. In addition, social distancing and the use of ventilated environments within the military site was guaranteed to have the population of interest gathered. The field team sent the questionnaire link to the military supervisors who were in shift the days we administered the instruments. When the military personnel were gathered, the supervisors were in charge of distributing virtually the instrument to all the sample, through internal WhatsApp groups. First, we requested the virtual filling out of their informed consent and then the filling out of the self-administered questionnaires for an average time of 20 min.

### Analysis plan

The univariate analysis showed absolute and relative frequencies of the categorical variables. For numerical variables, the distribution of the data has been evaluated in each variable before selecting the appropriate measure of central tendency. In cases where the data was normally distributed, the mean and standard deviation was used as the measure of central tendency. On the other hand, in cases where the data does not follow a normal distribution, the median and 25th -75th percentile was used as the measure of central tendency to avoid outliers affecting the interpretation of the results.

For the bivariate analysis between the outcomes of interest (depression and anxiety) and covariates, it was useful to use the Chi-squared test after assessing the assumption of expected frequencies.

Additionally, to explore the factors associated with depression and anxiety, we used generalized linear models with Poisson family distribution, log-link function and robust variances. We estimated prevalence ratios (PR) as a measure of association along with confidence intervals at 95% in the simple and multiple models. In the multiple model, we only included the variables that were statistically significant (p < 0.05) in the simple model. Interaction and collinearity among the covariates of interest were evaluated.

### Ethical aspects

The protocol study was approved by the Ethics Committee of the Universidad San Martin de Porres (File N°269-2021-CIEI-FMH-USMP). Informed consent was obtained from the military members before participating in the research. Confidentiality of military personnel was preserved at all times, and their participation was voluntary, without any type of obligation by their superiors.

## Results

### Characteristics of the study population

We analyzed the data of 615 military personnel that participated in the survey. Of them, 93.7% were male; the median age was 22 years old, with the 25th and 75th percentiles being 19 and 32 years, respectively; 73.3% were single; and 69.9% reported being Catholic. In addition, 5.6% were type 1 obese (the mean BMI was 24.68 ± 3.35) and 9.1% mentioned that they had arterial hypertension. Also, 4.2% informed that they had family history of mental health; and 8.0% affirmed that they had sought for mental health due to the pandemic. Moreover, 19.2% experienced fear of COVID-19 (the median score was 8, with the 25th and 75th percentiles being 7 and 14, respectively); 3.0% suffered from moderate insomnia (the median score for insomnia was 3, with the 25th and 75th percentiles being 0 and 7, respectively); and 43.2% had a high level of resilience (the mean resilience score was 22.18 ± 14.96). Table 1.

**Table 1:** Participant characteristics (n = 615)

Characteristics	N (%)
Age (years)*	22(19–32)
Gender	
Female	39( 6.3)
Male	576( 93.7)
Marital status	
Single	451( 73.3)
Married	143( 23.3)
Cohabiting	13( 2.1)
Divorced	8( 1.3)
Religion	
None	90( 14.6)
Catholic	430( 69.9)
Non-Catholic	95( 15.5)
Having children	169( 27.5)
Alcoholism	107( 17.4)
Smoking	40( 6.5)
Comorbidity	
Hypertension	56( 9.1)
Diabetes	11( 1.8)
BMI (numerical)**	24.68 ± 3.35
BMI (categorized)	
Underweight	9( 1.3)
Normal	400( 57.7)
Overweight	239( 34.5)
Obesity Type 1	39( 5.6)
Obesity Type 2	5( 0.7)
Obesity Type 3	1( 0.1)
Personal mental health history	7( 1.1)
Family mental health history	26( 4.2)
Seeking mental health support	49( 8.0)
Trust in the government to manage COVID-19	
Yes	334( 54.3)
No	281( 45.7)
Working time	
1 to 6 months	154( 25.6)
7 to 12 months	100( 16.6)
13 to18 months	128( 21.3)
19 months or more	219( 36.4)
Food insecurity	
No	316( 51.4)
Yes	299( 48.6)
Insomnia (numerical)*	3 (0–7)
Insomnia (categorized)	
Absence of clinical insomnia	436( 77.0)
Subclinical insomnia	104( 18.4)
Moderate clinical insomnia	17( 3.0)
Severe clinical insomnia	9( 1.6)
Level of physical activity	
Low	64( 10.4)
Moderate	39( 6.3)
High	512( 83.3)
Resilience (numerical)**	22.18 ± 14.96
Resilience (categorized)	
Low	333( 56.8)
High	253( 43.2)
Fear of COVID-19 (numerical)*	8 (7–14)
Fear of COVID-19 (categorized)	
No	424( 80.8)
Yes	101( 19.2)
Anxiety symptoms (numerical)*	0 (0–4)
Anxiety symptoms (categorized)	
No anxiety	464( 78.0)
Mild	86( 14.5)
Moderate	30( 5.0)
Severe	15( 2.5)
Depression symptoms (numerical)*	1 (0–6)
Depression symptoms (categorized)	
None	423( 70.2)
Mild	115( 19.1)
Moderate	45( 7.5)
Moderate-severe	12( 2.0)
Severe	8( 1.3)

*Median (percentile 25th - percentile 75th )

**Mean ± standard deviation

### Depression (PHQ-9) and anxiety (GAD-7) symptoms in military personnel

The median score for anxiety symptoms was 0, with the 25th and 75th percentiles being 0 and 4, respectively. The median score for depression symptoms was 1, with the 25th and 75th percentiles being 0 and 6, respectively. The prevalence of depression and anxiety symptoms was 29.9% and 22%, respectively. According to the level of severity, most of them presented with mild depressive symptoms (19.1%) and anxiety symptoms (14.5%). Table 1.

### Factors associated with depression and anxiety symptoms in the bivariate analysis

The bivariate analysis evidenced statistically significant differences between depression and anxiety *symptoms* and the variables of interest. Military personnel with a high level of resilience had a lower frequency of depression *symptoms* (19% vs. 37.5%; p < 0.001) and anxiety *symptoms* (11.1 vs. 29.7%; p < 0.001), in comparison with military members with a low level of resilience. In addition, *symptoms* of depression frequency (56.4% vs. 22.9%; p < 0.001) and anxiety *symptoms*(56.4% vs. 13.0%) was superior in military members with fear of COVID-19, in comparison with those who reported no fear of COVID-19. Military personnel with a high level of resilience had a lower frequency of depression (100% vs. 17.7%) and anxiety (100% vs. 10.6%), compared with those without insomnia. Table 2.

**Table 2:** **Factors associated with depression and anxiety** symptoms **in military personnel on the first-line of defense against COVID-19, bivariate analysis**

Variables	*Depression* symptoms		p*	*Anxiety* symptoms		p*
	No (n = 423)	Yes (n = 180)		No (n = 464)	Yes (n = 131)	
	n(%)	n(%)		n(%)	n(%)	
Age (years)***	22(19–33)	22(19-29.5)	0.394**	22(19–32)	23(19–30)	0.846**
Gender			0.552			0.976
Female	29( 74.4)	10( 25.6)		28( 77.8)	8( 22.2)	
Male	394( 69.9)	170( 30.1)		436( 78.0)	123( 22.0)	
Marital status			**0.039**			0.108
Single	297( 67.0)	146( 33.0)		333( 76.4)	103( 23.6)	
Married	111( 79.9)	28( 20.1)		117( 84.8)	21( 15.2)	
Cohabiting	9( 69.2)	4( 30.8)		9( 69.2)	4( 30.8)	
Divorced	6( 75.0)	2( 25.0)		5( 62.5)	3( 37.5)	
Religion			0.518			0.494
None	60( 68.2)	28( 31.8)		66( 75.9)	21( 24.1)	
Catholic	301( 71.5)	120( 28.5)		329( 79.3)	86( 20.7)	
Non-Catholic	62( 66.0)	32( 34.0)		69( 74.2)	24( 25.8)	
Having children	124( 74.7)	42( 25.3)	0.132	130( 79.8)	33( 20.3)	0.522
Alcoholism	67( 64.4)	37( 35.6)	0.161	74( 73.3)	27( 26.7)	0.209
Smoking	19( 50.0)	19( 50.0)	**0.005**	20( 52.6)	18( 47.4)	**< 0.001**
Comorbidity						
Hypertension	36( 65.5)	19( 34.6)	0.425	39( 70.9)	16( 29.1)	0.184
Diabetes	6( 54.6)	5( 45.5)	0.254	7( 63.6)	4( 36.4)	0.246
BMI (categorized)			0.546			0.674
Underweight/normal	246( 69.1)	110( 30.9)		273( 77.8)	78( 22.2)	
Overweight	145( 73.2)	53( 26.8)		157( 80.1)	39( 19.9)	
Obesity	27( 67.5)	13( 32.5)		29( 74.4)	10( 25.6)	
Personal mental health history			**0.016**			**0.024**
No	421( 70.6)	175( 29.4)		461( 78.4)	127( 21.6)	
Yes	2( 28.6)	5( 71.4)		3( 42.9)	4( 57.1)	
Family mental health history			**< 0.001**			**0.007**
No	416( 72.0)	162( 28.0)		450( 79.0)	120( 21.1)	
Yes	7( 28.0)	18( 72.0)		14( 56.0)	11( 44.0)	
Seeking mental health support			0.227			0.107
No	393( 70.8)	162( 29.2)		431( 78.8)	116( 21.2)	
Yes	30( 62.5)	18( 37.5)		33( 68.8)	15( 31.3)	
Trust in the government to manage COVID-19			0.051			0.064
Yes	241( 73.5)	87( 26.5)		262( 80.9)	62( 19.1)	
No	182( 66.2)	93( 33.8)		202( 74.5)	69( 25.5)	
Working time			**< 0.001**			**< 0.001**
1 to 6 months	101( 67.3)	49( 32.7)		114( 76.5)	35( 23.5)	
7 to 12 months	64( 65.3)	34( 34.7)		68( 69.4)	30( 30.6)	
13 to 18 months	74( 58.3)	53( 41.7)		84( 67.2)	41( 32.8)	
19 months or more	174( 81.3)	40( 18.7)		188( 89.5)	22( 10.5)	
Food insecurity			**0.001**			0.145
No	236( 76.4)	73( 23.6)		246( 80.4)	60( 19.6)	
Yes	187( 63.6)	107( 36.4)		218( 75.4)	71( 24.6)	
Insomnia			**< 0.001**			**< 0.001**
Absence of clinical insomnia	359( 82.3)	77( 17.7)		390( 89.5)	46( 10.6)	
Subclinical insomnia	36( 34.6)	68( 65.4)		49( 47.1)	55( 52.9)	
Moderate clinical insomnia	4( 23.5)	13( 76.5)		5( 29.4)	12( 70.6)	
Severe clinical insomnia	0( 0.0)	9( 100.0)		0( 0.0)	9( 100.0)	
Level of physical activity			0.517			0.128
Low	41( 64.1)	23( 35.9)		44( 68.8)	20( 31.3)	
Moderate	27( 69.2)	12( 30.8)		29( 74.4)	10( 25.6)	
High	355( 71.0)	145( 29.0)		391( 79.5)	101( 20.5)	
Resilience			**< 0.001**			**< 0.001**
Low	208( 62.5)	125( 37.5)		234( 70.3)	99( 29.7)	
High	205( 81.0)	48( 19.0)		225( 88.9)	28( 11.1)	
Fear of COVID-19			**< 0.001**			**< 0.001**
No	327( 77.1)	97( 22.9)		369( 87.0)	55( 13.0)	
Yes	44( 43.6)	57( 56.4)		44( 43.6)	57( 56.4)	

*P-value of categorical variables calculated with the Chi-squared test

** P-value of categorical/numerical variables calculated with the U test (Mann-Whitney)

*** Median - interquartile range

### Factors associated with depression and anxiety symptoms in the simple and multiple regression model analysis

We found that married military members had a lower prevalence of depression *symptoms* in comparison with the single ones (PR: 0.63; 95% IC: 0.42–0.94). In addition, the prevalence of depression *symptoms* was higher in military personnel that reported having a relative with mental health problems (PR: 2.16; 95% IC: 1.52–3.08) and those who had experienced food insecurity (PR: 1.48; 95% IC: 1.16–1.89). Military personnel with insomnia and fear of COVID-19 had 171% (PR: 2.71; 95% IC: 2.04–3.59) and 48% (PR: 1.48; 95% IC: 1.15–1.91) higher prevalence of depression *symptoms*, respectively, Additionally, having a high level of resilience was associated with a lower prevalence of depression *symptoms* (PR: 0.65; 95% IC: 0.48–0.88). Table 3.

**Table 3:** **Factors associated with depression and anxiety** symptoms **in military personnel on the first-line of defense against COVID-19, bivariate analysis simple and multiple regression analysis**

Characteristics		*Depression* symptoms						*Anxiety* symptoms					
		Simple regression			Multiple regression			Simple regression			Multiple regression		
		PR	95% CI	p*	PR	95% CI	p*	PR	95% CI	p*	PR	95% CI	p*
Age (years)		0.99	0.98-1.00	0.166				1.00	0.98–1.01	0.564			
Gender													
	Female	Ref.						Ref.					
	Male	1.18	0.68–2.04	0.564				0.99	0.53–1.86	0.976			
Marital status													
	Single	Ref.			Ref.			Ref.			Ref.		
	Married	0.61	0.43- -0-87	**0.007**	0.63	0.42–0.94	**0.024**	0.64	0.42–0.99	**0.044**	0.66	0.40–1.09	0.103
	Cohabiting	0.93	0.41–2.13	0.871	0.86	0.34–2.15	0.745	1.30	0.57-3.00	0.534	1.35	0.55–3.32	0.518
	Divorced	0.76	0.23–2.54	0.654	1.17	0.44–3.13	0.754	1.59	0.64–3.95	0.320	2.43	0.86–6.88	0.095
Religion													
	None	Ref.						Ref.					
	Catholic	0.90	0.64–1.26	0.528				0.86	0.57–1.30	0.474			
	Non-Catholic	1.07	0.71–1.62	0.750				1.07	0.64–1.78	0.796			
Having children		0.80	0.60–1.08	0.142				0.89	0.63–1.27	0.525			
Alcoholism		1.24	0.93–1.67	0.149				1.27	0.88–1.83	0.200			
Smoking		1.75	1.09–2.82	**0.020**	0.95	0.65–1.38	0.771	2.33	1.42–3.84	**0.001**	0.98	0.65–1.49	0.931
Comorbidity													
	Hypertension	1.18	0.80–1.73	0.412				1.37	0.88–2.13	0.168			
	Diabetes	1.54	0.79–2.97	0.201				1.67	0.75–3.71	0.206			
BMI (categorized)													
	Underweight/normal	Ref.						Ref.					
	Overweight	0.87	0.66–1.14	0.312				0.90	0.64–1.26	0.527			
	Obesity	1.05	0.66–1.69	0.834				1.15	0.65–2.04	0.622			
Personal mental health history													
	No	Ref.			Ref.			Ref.			Ref.		
	Yes	2.43	1.50–3.95	**< 0.001**	1.03	0.61–1.75	0.902	2.65	1.37–5.12	**0.004**	0.95	0.47–1.90	0.873
Family mental health history													
	No	Ref.			Ref.			Ref.			Ref.		
	Yes	2.57	1.95–3.39	**< 0.001**	2.16	1.52–3.08	**< 0.001**	2.09	1.31–3.35	**0.002**	1.28	0.76–2.16	0.359
Seeking mental health support													
	No	Ref.						Ref.					
	Yes	1.28	0.87–1.89	0.205				1.47	0.94–2.31	0.091			
Trust in the government to manage COVID-19													
	Yes	Ref.						Ref.					
	No	1.27	0.99–1.63	0.052				1.33	0.98–1.80	0.065			
Working time													
	1 to 6 months	Ref.			Ref.			Ref.			Ref.		
	7 to 12 months	1.06	0.74–1.52	0.740	1.00	0.69–1.43	0.984	1.30	0.86–1.98	0.212	0.94	0.63–1.39	0.744
	13 to18 months	1.28	0.94–1.74	0.120	1.22	0.89–1.67	0.216	1.40	0.95–2.05	0.088	0.94	0.64–1.39	0.761
	19 months or more	0.57	0.40–0.82	**0.003**	0.81	0.56–1.17	0.266	0.45	0.27–0.73	**0.001**	0.52	0.34–0.81	**0.004**
Food insecurity													
	No	Ref.			Ref.			Ref.					
	Yes	1.54	1.20–1.98	**0.001**	1.48	1.16–1.89	**0.001**	1.25	0.92–1.70	0.146			
Insomnia													
	No	Ref.			Ref.			Ref.			Ref.		
	Yes	3.92	3.11–4.95	**< 0.001**	2.71	2.04–3.59	**< 0.001**	5.54	4.07–7.55	**< 0.001**	3.32	2.27–4.87	**< 0.001**
Level of physical activity													
	Low	Ref.						Ref.			Ref.		
	Moderate	0.86	0.48–1.52	0.596				0.82	0.43–1.57	0.549	1.33	0.74–2.38	0.339
	High	0.81	0.57–1.15	0.236				0.66	0.44–0.98	**0.041**	1.13	0.79–1.62	0.505
Resilience													
	Low	Ref.			Ref.			Ref.			Ref.		
	High	0.51	0.38–0.68	**< 0.001**	0.65	0.48–0.88	**0.005**	0.37	0.25–0.55	**< 0.001**	0.50	0.33–0.77	**0.001**
Fear of COVID-19													
	No	Ref.			Ref.			Ref.			Ref.		
	Yes	2.47	1.93–3.15	**< 0.001**	1.48	1.15–1.91	**0.002**	4.35	3.22–5.88	**< 0.001**	2.43	1.73–3.40	**< 0.001**

*P-values obtained with Generalized Linear Models (GLM), *Poisson* family, log-link function, robust variance

In regard to anxiety *symptoms*, working more than 18 months since the beginning of the COVID pandemic (PR: 0.52; 95% IC: 0.34–0.81) and a high level of resilience (PR: 0.50; 95% IC: 0.33–0.77) diminished the prevalence of anxiety *symptoms*. On the other hand, the prevalence of anxiety *symptoms* was 232% superior in military personnel with insomnia, in comparison with those who did not have insomnia (PR: 3.32; 95% IC: 2.27–4.87). Additionally, the military members with fear of COVID-19 had 143% higher prevalence of anxiety *symptoms*, in comparison with those who did not have that fear (PR: 2.43; 95% IC: 1.73–3.40).

## Discussion

### Prevalence of depressive symptoms

Almost three out of ten participants (29.9%) had depressive symptoms. Similarly, before the pandemic, the study by Bareis and Mezuck identified that 24% of American military members had depression [45]. Likewise, it was similar to the results of a meta-analysis conducted by Moradi et al., who reported a prevalence of depression of 23% in active military forces [12]. However, this is superior to what was found before the COVID-19 pandemic by Lopez et al. in Colombian military members, as a prevalence of 8.8% was found [46]. This was similar to Britton et al., as they found that the American participants with a background of military service had a prevalence of depression of 13%, in comparison with the ones who did not have military activity [47]. That is superior to what was reported during the pandemic by Caycho et al. who found a prevalence value of 19.6% in Peruvian police officers [48], and by Sudom and Lee, as they estimated a 14.3% value of depression in Canadian soldiers [49]. However, it is inferior to what was described by Sapkota et al. that informed 42.3% prevalence in active military personnel before the pandemic [50]. Moreover, Smetana et al. identified that the prevalence of depression in American military members varied over the course of the pandemic up to 40.4% [51], and Bond et al. with a prevalence value of 41.9% before COVID-19 in active American military personnel [52]. This result could be due to the real risk of infection and the incontrollable fear of death due to the pandemic [53] and for the different situations that the participants faced such as being away of the family and being in quarantine [12].

### Prevalence of anxiety symptoms

In the total sample, 22% had anxiety symptoms. This is similar to what was reported by Stevelink et al. that identified 21.9% of British military personnel informed anxiety symptoms before the pandemic [54]. Nevertheless, that is superior to what was reported during the pandemic by Caycho et al., who found a prevalence of 17.3% in Peruvian police officers [48], and to what was described by Finnegan and Randles that reported a 15% prevalence among British military veterans [55]. In addition, it differs from what was found by Sudom and Lee in a study conducted at the beginning of the pandemic, in which a lower prevalence was found (14.5%) [49]. Moreover, Kessler et al. reported a prevalence of 5.7% among American military personnel before the pandemic [56]. This result could be due to the risk of infection, the unpredictability of virus dissemination and uncertainty of everyday life [53]. It also could be due to the fact that during the pandemic they performed their activities with limited resources (inadequate protective equipment) and they were exposed to a higher risk of contagion of the disease, which led to stress increase [48] and, hence, anxiety. Additionally, the prevalence of depressive symptoms could be explained by pre-pandemic factors (low economic level, educational level, and psychiatric history) and post-pandemic factors (the level of resilience and social support) [57]. Situations that could fluctuate in the course of the pandemic and that impacted each of the disorders.

### Factors associated with depression

Being married reduced depression prevalence. This is similar to what was reported by Britton et al., who identified that military personnel who lived with their partners had a lower risk of depression [47]. In addition, it is consistent with what was reported by Smertana et al., who demonstrated that single people had up to 76.8% of depression prevalence [51]. Sudon and Lee reported that not having children, as single couples or not married had negative effects on mental health (depression) [49]. Nevertheless, it differs from what was found by Bulloch et al. who identified that depression was lower in widows, divorced, and separated people [58]. This finding could be explained by the associations between the variables showed variations according to the age group of the people. In addition, there is a relationship in regard to economic resources as married people had fewer economic problems than the single ones; this fact lead to better mental wellbeing [59]. Moreover, it was identified that an adequate family functioning through expressiveness, relationships, cohesion, and life events related with family are associated with a lower risk of PTSD [60]; we can highlight that family functioning was not measured in this study.

The participants that reported having a family member with mental health problems had 116% higher prevalence of depression. This result is similar to what was reported by Colvin et al., who found a 2.2 times higher probability for those people who had family history of depression in comparison with those without family history (OR = 2.24, 95% CI:1.17–4.29, p = 0.02). We can highlight that this study was conducted in a different population and context [61]. This finding could be explained because a relative with a previous mental health history produces a negative impact on the mental sphere of family members, particularly in regard to depression [62]. The found relationship could also be explained by predisposing neurobiological factors that might be inherited for depression [63]. The significant evidence shows that people with family history of depression had smaller prefrontal cortex and hippocampal volumes as well as thinner gray matter in extensive areas of the right hemisphere, in comparison with subjects without first-degree family history of depression [64].

Food insecurity (FI) increased the prevalence of depression by 48%. This is similar to Cohen et al., who found that military veterans from the USA with food insecurity had a prevalence of 36.2% of having a diagnosis of depression during the pre-pandemic context [65]. Similarly, Brostow et al., reported a significant positive association between FI and depression in military veterans from the USA [66]. In addition, Wang et al. reported a positive association between FI and depression in military veterans (AOR = 3.00, 95% CI: 2.60–3.46); it is important to highlight that this study was not conducted in the context of the COVID-19 pandemic [67]. Nevertheless, a bidirectional relationship has been evidenced according to some studies [68]. FI behaves as a confounding variable because its association with mental health disorders is large. [69–71] FI in our country is of great importance due to its high prevalence during the context of the pandemic with 37.1% in the general population. [71], in addition, FI has been shown to predispose to a 257% and 253% risk of anxiety and depression, respectively. [69].According to scientific evidence, FI is associated with mental disorders such as depression and also increases the appearance of its symptoms. This occurs due the fact that FI can affect nutritional status and cause psychological suffering due to the incapacity to feed themselves or their families; in addition, intake deficiency as a result of food insecurity can affect negatively brain functioning and compromise cognition and emotions [72].

Having insomnia increased the prevalence of depression by 171%. This is similar to what was reported by Sudom and Lee, who found that enough sleep as a healthy behavior reduced (OR = 0.34; 95% CI: 0.30–0.38) the probability of having depression in Canadian military members during the pandemic [49]. Similarly, a pre-pandemic study identified that the different characteristics of insomnia (prolonged sleep latency, low sleep efficiency and short sleep duration) were associated with a higher frequency of depressive symptoms [73]. Moreover, it is consistent with what was described by Choi et al., who found that those who had insomnia symptoms reported more depressive symptoms (64.7%) before the pandemic in Korea [74], which evidences that insomnia is associated with physiological aspects of depression such as appetite, suicide and psychomotor symptoms [75]. This association could be explained by the deficiency of glutamate, which plays an important role in depression and sleep regulation, and the reduction of melatonin in addition to cataloguing insomnia as a modifiable factor for insomnia development [76].

The participants with a high level of resilience decreases depression prevalence by 35%. This result is similar to what was reported by Valladares et al., who found that having a high level of resilience was associated, in an independent form, with a lower frequency of depression symptoms (PR = 0.53; 95% IC = 0.42–0.68) in general population of Peru during the pandemic [77]. In addition, Mitchell et al. identified that high levels of resilience led to a lower level of depression in Irish people during the pandemic [18]. Bartone and Homish identified that military veterans with resilience patterns with the use of avoidance coping strategies developed lower levels of depression [78]. To et al., identified that resilience had a positive significant association with depression in Australia during the pandemic [79]. However, it differs from Karaşar y Canli’s study, who found a medium and negative correlation between resilience and depression during the COVID-19 pandemic in Turkey [80]. The association found could be explained because resilience through indicators such as life satisfaction, subjective wellbeing and hope reduces the prevalence of depressive symptoms [81].

Fear of COVID was associated with a 48% higher depression prevalence. This is similar to what was reported by Yuan et al. in their meta-analysis, in which it was identified that having great concern due to the pandemic aggravated the symptoms of depression. We can highlight that the study focused on general population [82]. In relation to this association, we did not find another similar study. This finding could be explained by uncertainty, information overload, and isolation as a product of the pandemic [83]. In addition, there could be hypersensitivity of the fear network such as increased amygdala and decreased hippocampal volume as drivers of the development of depression [84]. However, this association of variables is not well documented.

### Factors associated with anxiety

Having worked for 19 months on the first line of defense against COVID-19 represented a 48% reduction in the prevalence of anxiety. We did not find studies with similar results. This is contrary to what was reported by Yun et al., who reported that health personnel (who were on the first line) had a higher level of anxiety (35.9%, 95% CI: 31.5–40.3), in comparison with other populations included in that study [82]. Also, it differs from what was described by Magnavita et al. in general population, who proved that the time of service would lead to a higher level of depression, given that the symptoms of depression increased from 49.9 to 62.5% in the first and second wave, respectively [85]. This finding could be explained due to the fact that during the pandemic the people who were on the first line such as health personnel, military personnel, among others, were under higher levels of stress, but had a good management of knowledge and coping strategies to help alleviate mental health problems such as anxiety [82]. Another probable explanation for the fact that military personnel with more time of service on the first line of defense against COVID reported a lower level of frequency of anxiety symptoms is that they could be more adapted to health emergency situations, in contrast with the military personnel who were recently beginning their work against COVID.

The prevalence of anxiety increased by 232% in participants with insomnia. This is similar to what was reported by Sudom and Lee, who identified that sleeping enough was associated with a lower probability (OR:0.35) of having anxiety [49]. Bello et al. demonstrated, through a meta-analysis, that chronic and/or existent comorbidities such as insomnia behaved as main predictors of anxiety in general population [86]. The probable explanation for this association could be due to a physiopathological mechanism of cortisol shared by both disorders. People with anxiety exhibit a blunted cortisol awakening response and higher total cortisol production, just as people with sleep disturbance (insomnia) have dysregulated diurnal cortisol. Therefore, over time, the subsequent effects of dysregulated cortisol can mediate in the relationship between the symptoms of anxiety and insomnia [87]. In addition, people with insomnia usually worry excessively due to their sleep and consequences of sleep deprivation, because of this concern, this would cause an extreme state of anxiety [88].

Having a high level of resilience reduced the prevalence of anxiety by half. This is similar to what was reported by Ran et al. in general population during the first wave of the pandemic in China, as these authors found a positive correlation between a high level of resilience and anxiety [89]. Similarly, To et al. reported a positive significant association between resilience and anxiety during the pandemic in Australian general population [79]. Skalsi et al. reported that the correlation between resilience and anxiety is mediated by persistent thinking about COVID-19 in Polish adult general population [90] and concluded that resilience acts by mitigating the development of a mental disease. Ligeza et al. identified that among members of the U.S. National Guard, resilience is a protective factor against the development of anxiety during the pandemic [91]. This association could be due to the fact that higher levels of resilience through greater access to resources and greater family support condition the development of positive emotions and greater social adaptability [53], situations that decrease anxiety.

Fear of COVID-19 increased the prevalence of anxiety by 143%. This is similar to what was reported by Kalin, who showed that fear of contracting COVID-19, becoming seriously ill and dying, predisposed the development of anxiety in the general US population [92]. This is similar to what was reported by the meta-analysis of Yuan et al., who identified that having great concern about the pandemic aggravated anxiety symptoms in the general population [82]. This finding could be explained by evidence of the existence of the fear network, where the amygdala plays the most important role in as a response to the threat that subsequently leads to the development of anxiety [84]. However, this association of variables is not well documented.

### Implications of the findings for mental health

These preliminary results will be useful for the implementation and improvement of mental health problems in military departments due to the fact that during the pandemic many limitations were evidenced such as little accessibility to hospitals due to the increase in COVID-19 cases. In addition, this population performs high-risk operations in response to emergencies and disasters [9]; therefore, preserving good mental health is essential in this work group by implementing a contingency plan for future natural disasters or pandemics. It is also important to establish an adequate mental health monitoring in order to avoid the persistence of depressive or anxiety symptoms, or the worsening of their situation which could lead to suicidal ideation or attempts, among others.

### Limitations and strengths

Our study has some limitations. First, selection bias, due to the fact that we used non-probability snowball sampling. Our findings are considered preliminary and future studies should improve the sampling of the study population. In addition, the cross-sectional design does not allow the identification of causal relationships between the study variables. Some confounding variables were not measured such as compliance with public health guidelines (use of masks and face shield, frequent hand washing, avoiding crowded meetings, among others) [13], knowing whether the participants had at any time a positive PCR test or antigen for SARS-CoV-2 [51], and military rank [18]. Another limitation is that the non-probabilistic sampling method limits the inference of the data. Despite these limitations, this study provides novel information with the use of validated instruments to elicit a variety of characteristics that may trigger mental problems. Also, we were unable to consider among the exclusion criteria those persons with a previous diagnosis of a mental health disorder or substance abuse. The strengths of our study lie in the sample size, the instruments used that can be administered through an interview or be self-administered; and also, to our knowledge, it is the first study conducted in military personnel during COVID-19 that assesses symptoms of depression and anxiety. Our findings are relevant because they provide the necessary information to build the foundations for the development of additional and better designed research, which will finally promote the design of effective preventive and protective strategies for military population, who are a fundamental part of the state in the face of any eventuality.

## Conclusions

We found a symptomatology prevalence of 29.9% for depression and 22% for anxiety. In regard to the attenuating factors of depression, these were being married and having resilience; and having a relative with mental health problems, food insecurity, insomnia, and fear of COVID-19 were found among the aggravating factors. Finally, anxiety increased through working time, insomnia, and fear of COVID-19.

## Data Availability

The dataset generated and analyzed during the current study is not publicly available due data sharing restriction stablished by the ethics committee but is available from the corresponding author on reasonable request.

## References

[1] Fernandez-Canani MA, Burga-Cachay SC, Valladares-Garrido MJ (2022). Association between Family Dysfunction and post-traumatic stress disorder in School students during the second COVID-19 epidemic Wave in Peru. Int J Environ Res Public Health.

[2] Ponce VV, Garrido MV, Peralta CI (2020). Factores asociados al afrontamiento psicológico frente a la COVID-19 durante el periodo de cuarentena. Rev Cuba Med Mil.

[3] Hettich N, Entringer TM, Kroeger H (2022). Impact of the COVID-19 pandemic on depression, anxiety, loneliness, and satisfaction in the german general population: a longitudinal analysis. Soc Psychiatry Psychiatr Epidemiol.

[4] Ayhan-Balik CH, Karakaya S, Kutlu FY (2022). Factors affecting anxiety and depression during the first wave of the COVID-19 pandemic: a cross-sectional study of three different populations. Egypt J Neurol Psychiatry Neurosurg.

[5] Xiong J, Lipsitz O, Nasri F (2020). Impact of COVID-19 pandemic on mental health in the general population: a systematic review. J Affect Disord.

[6] Villarreal-Zegarra D, Copez-Lonzoy A, Vilela-Estrada AL, Huarcaya-Victoria J (2021). Depression, post-traumatic stress, anxiety, and fear of COVID-19 in the general population and health-care workers: prevalence, relationship, and explicative model in Peru. BMC Psychiatry.

[7] Hernández-Díaz Y, Genis-Mendoza AD, Ramos-Méndez M (2022). Mental Health Impact of the COVID-19 pandemic on Mexican Population: a systematic review. Int J Environ Res Public Health.

[8] Hernández-Díaz Y, Genis-Mendoza AD, Ramos-Méndez M (2022). Mental Health Impact of the COVID-19 pandemic on Mexican Population: a systematic review. Int J Environ Res Public Health.

[9] Valladares-Garrido MJ, Picón-Reátegui CK, Zila-Velasque JP, Grados-Espinoza P (2022). Prevalence and factors Associated with Insomnia in Military Personnel: a retrospective study during the second COVID-19 epidemic Wave in Peru. Healthcare.

[10] Guo X, Wu L, Yu X, Sun Z, Liu W (2020). Mental Health Care for Military Personnel in the COVID-19 epidemic. Mil Med.

[11] Asare-Doku W, Donnir GM, Ayuurebobi Ae-Ngibise K, Peprah J, Awuviry-Newton K, Acquah F (2021). Psychiatric Disorders among the military in West Africa: a systematic narrative review. Behav Sci Basel Switz.

[12] Moradi Y, Dowran B, Sepandi M (2021). The global prevalence of depression, suicide ideation, and attempts in the military forces: a systematic review and Meta-analysis of cross sectional studies. BMC Psychiatry.

[13] Adler AB, Gutierrez IA, Gomez SAQ (2022). US soldiers and the role of leadership: COVID-19, mental health, and adherence to public health guidelines. BMC Public Health.

[14] Sudom KA, Lee JEC (2022). Well-being of canadian Armed Forces members during the COVID-19 pandemic: the influence of positive health behaviours. Health Promot Chronic Dis Prev Can Res Policy Pract.

[15] Pritchard A, Dymond S (2022). Gambling problems and associated harms in United Kingdom Royal Air Force personnel. Addict Behav.

[16] Kotoulas AS, Karamanavis D, Lambrou G, Karanikas P. A pilot study of the depression, anxiety and stress in Greek military personnel during the first year of the COVID-19 pandemic. *BMJ Mil Health*. Published online July 2021:bmjmilitary-2021-001874. doi:10.1136/bmjmilitary-2021-001874 10.1136/bmjmilitary-2021-001874 34266974

[17] Hill ML, Nichter B, Na PJ et al. Mental health impact of the COVID-19 pandemic in U.S. military veterans: a population-based, prospective cohort study.*Psychol Med*. Published online2021:1–12. doi:10.1017/S0033291721002361 10.1017/S0033291721002361 PMC8245339 34120667

[18] Mitchell NA, McCauley M, O’Brien D, Wilson CE (2022). Mental health and resilience in the irish defense forces during the COVID-19 global pandemic. Mil Psychol.

[19] Osorio-Martínez M, Malca-Casavilca M, Condor-Rojas Y (2022). Factores asociados al desarrollo de estrés, ansiedad y depresión en trabajadores sanitarios en el contexto de la pandemia por COVID-19 en Perú. Arch Prev Riesgos Laborales.

[20] Chew NWS, Lee GKH, Tan BYQ (2020). A multinational, multicentre study on the psychological outcomes and associated physical symptoms amongst healthcare workers during COVID-19 outbreak. Brain Behav Immun.

[21] Campos-De La Cruz I, Burneo-Ramírez MC, Runzer-Colmenares FM, Campos-De La Cruz I, Burneo-Ramírez MC, Runzer-Colmenares FM (2021). Asociación entre salud mental y actitudes ante el confinamiento durante la pandemia COVID-19 en hospitales de Lima y Callao. Acta Médica Peru.

[22] Díaz-Vélez C, Failoc-Rojas VE, Valladares-Garrido MJ (2021). SARS-CoV-2 seroprevalence study in Lambayeque, Peru. June–July 2020. PeerJ.

[23] Díaz-Vélez C, Urrunaga-Pastor D, Romero-Cerdán A (2021). Risk factors for mortality in hospitalized patients with COVID-19 from three hospitals in Peru: a retrospective cohort study. F1000Research.

[24] Huarcaya-Victoria J, De-Lama-Morán R, Quiros M (2020). Propiedades psicométricas del Patient Health Questionnaire (PHQ-9) en estudiantes de medicina en Lima, Perú. Rev Neuro-Psiquiatr.

[25] Osorio-Martínez ML, Malca-Casavilca M, Condor-Rojas Y, Becerra-Bravo MA, Ramirez ER (2022). Factores asociados al desarrollo de estrés, ansiedad y depresión en trabajadores sanitarios en el contexto de la pandemia por COVID-19 en Perú. Arch Prev Riesgos Laborales.

[26] Alvarez EAC, Virú-Flores H, Alburqueque-Melgarejo J, et al. Validación del cuestionario sobre la salud del paciente–9 (PHQ-9) en internos de medicina humana de una universidad de referencia del Perú durante la pandemia COVID-19: validation of the Patient Health Questionnaire-9 (PHQ-9) in human medicine interns at a reference university in Peru during the COVID-19 pandemic. Rev Fac Med Humana. 2022;22(3). 10.25176/RFMH.v21i1.3179.

[27] Camargo L, Herrera-Pino J, Shelach S (2021). Escala de ansiedad generalizada GAD-7 en profesionales médicos colombianos durante pandemia de COVID-19: validez de constructo y confiabilidad. Rev Colomb Psiquiatr Published online.

[28] Morin CM, Belleville G, Bélanger L, Ivers H (2011). The Insomnia Severity Index: psychometric indicators to detect insomnia cases and evaluate treatment response. Sleep.

[29] Sierra JC, Guillén-Serrano V, Santos-Iglesias P (2008). [Insomnia Severity Index: some indicators about its reliability and validity on an older adults sample]. Rev Neurol.

[30] Fernandez-Mendoza J, Rodriguez-Muñoz A, Vela-Bueno A (2012). The spanish version of the Insomnia Severity Index: a confirmatory factor analysis. Sleep Med.

[31] Vargas Puello V, Alvarado Orellana S, Atalah Samur E (2013). Inseguridad alimentaria en adultos mayores en 15 comunas del gran Santiago: un tema pendiente. Nutr Hosp.

[32] Coates J, Swindale A, Bilinsky P. Escala del Componente de Acceso de la Inseguridad Alimentaria en el Hogar (HFIAS) para la Medición del Acceso a los Alimentos en el Hogar: Guía de Indicadores. Published online 2007.

[33] Teh L, Pirkle C, Furgal C, Fillion M, Lucas M (2017). Psychometric validation of the household food insecurity access scale among Inuit pregnant women from Northern Quebec. PLoS ONE.

[34] Naja F, Hwalla N, Fossian T, Zebian D, Nasreddine L (2015). Validity and reliability of the arabic version of the Household Food Insecurity Access Scale in rural Lebanon. Public Health Nutr.

[35] Knueppel D, Demment M, Kaiser L (2010). Validation of the Household Food Insecurity Access Scale in rural Tanzania. Public Health Nutr.

[36] Psaki S, Bhutta ZA, Ahmed T (2012). Household food access and child malnutrition: results from the eight-country MAL-ED study. Popul Health Metr.

[37] Mantilla Toloza SC, Gómez-Conesa A (2007).

[38] Cuestionario internacional de actividad física (IPAQ). Published online 2022. https://www.juntadeandalucia.es/export/drupaljda/salud_5af95872aeaa7_cuestionario_actividad_fisica_ipaq.pdf

[39] Cancela Carral JM, Lago Ballesteros J, Ayán Pérez C, Mosquera Morono MB (2016). Análisis de fiabilidad y validez de tres cuestionarios de autoinforme para valorar la actividad física realizada por adolescentes españoles. Gac Sanit.

[40] Crespo M, Fernández Lansac V, Soberón C. Adaptación española de la “Escala de resiliencia de Connor-Davidson” (CD-RISC) en situaciones de estrés crónico.*Psicol Conduct*. Published online2014:219–238.

[41] Campbell-Sills L, Stein MB (2007). Psychometric analysis and refinement of the Connor-davidson Resilience Scale (CD-RISC): validation of a 10-item measure of resilience. J Trauma Stress.

[42] Bernaola Ugarte AD, Garcia Garcia M, Martinez Campos N, Ocampos Madrid M, Livia J. Validez y confiabilidad de la escala breve de Resiliencia Connor-Davidson (CD-RISC 10) en estudiantes universitarios de Lima Metropolitana. *Cienc Psicológicas*. Published online May. 2022;30. 10.22235/cp.v16i1.2545.

[43] Ahorsu DK, Lin CY, Imani V, Saffari M, Griffiths MD, Pakpour AH (2022). The fear of COVID-19 scale: development and initial validation. Int J Ment Health Addict.

[44] Huarcaya-Victoria J, Villarreal-Zegarra D, Podestà A, Luna-Cuadros MA (2022). Psychometric Properties of a spanish version of the fear of COVID-19 scale in General Population of Lima, Peru. Int J Ment Health Addict.

[45] Bareis N, Mezuk B (2016). The relationship between childhood poverty, military service, and later life depression among men: evidence from the Health and Retirement Study. J Affect Disord.

[46] López SMA, Bedoya Mejía S, Henao Valencia MC (2020). Prevalencia de depresión en soldados regulares de un batallón de una ciudad colombiana, 2017. Rev Médica Risaralda.

[47] Britton PC, Bossarte RM, Lu N (2011). Prevalence, correlates, and symptom profiles of depression among men with a history of military service. Soc Psychiatry Psychiatr Epidemiol.

[48] Caycho-Rodríguez T, Carbajal-León C, Vilca LW (2020). COVID-19 y salud mental en policías peruanos: resultados preliminares. Acta Médica Peru.

[49] Sudom KA, Lee JEC (2022). Well-being of canadian Armed Forces members during the COVID-19 pandemic: the influence of positive health behaviours. Health Promot Chronic Dis Prev Can Res Policy Pract.

[50] Sapkota N, Tiwari A, Kunwar M, Manandhar N, Khatri B (2022). Depression among Armed Police Force Soldiers serving in a police headquarter: a descriptive cross-sectional study. JNMA J Nepal Med Assoc.

[51] Smetana C, Patel D, Stahlman S, Chauhan A, Wells N, Ying S (2022). COVID-19 and depressive symptoms among active component U.S. service members, January 2019-July 2021. MSMR.

[52] Bond GR, Al-Abdulmunem M, Drake RE (2022). Transition from Military Service: Mental Health and Well-being among Service Members and Veterans with Service-connected disabilities. J Behav Health Serv Res.

[53] Song S, Yang X, Yang H (2021). Psychological resilience as a protective factor for depression and anxiety among the Public during the outbreak of COVID-19. Front Psychol.

[54] Stevelink SAM, Jones M, Hull L (2018). Mental health outcomes at the end of the british involvement in the Iraq and Afghanistan conflicts: a cohort study. Br J Psychiatry J Ment Sci.

[55] Finnegan A, Randles R (2022). Prevalence of common mental health disorders in military veterans: using primary healthcare data. BMJ Mil Health Published online.

[56] Kessler RC, Heeringa SG, Stein MB (2014). Thirty-day prevalence of DSM-IV mental disorders among nondeployed soldiers in the US Army: results from the Army study to assess risk and resilience in servicemembers (Army STARRS). JAMA Psychiatry.

[57] Friedman MJ, Schnurr PP, McDonagh-Coyle A (1994). Post-traumatic stress disorder in the military veteran. Psychiatr Clin North Am.

[58] Bulloch AGM, Williams JVA, Lavorato DH, Patten SB (2017). The depression and marital status relationship is modified by both age and gender. J Affect Disord.

[59] Grundström J, Konttinen H, Berg N, Kiviruusu O (2021). Associations between relationship status and mental well-being in different life phases from young to middle adulthood. SSM - Popul Health.

[60] McDonald CC, Deatrick JA (2011). The role of Family Phenomena in Posttraumatic stress in Youth. J Child Adolesc Psychiatr Nurs Off Publ Assoc Child Adolesc Psychiatr Nurses Inc.

[61] Colvin A, Richardson GA, Cyranowski JM, Youk A, Bromberger JT (2017). The role of Family History of Depression and the menopausal transition in the development of major depression in midlife women: study of women’s Health across the Nation Mental Health Study (SWAN MHS). Depress Anxiety.

[62] Wittenberg E, Saada A, Prosser LA (2013). How illness affects family members: a qualitative interview survey. The Patient.

[63] Serna-Arbeláez D, Terán-Cortés CY, Vanegas-Villegas AM et al. Depresión y funcionamiento familiar en adolescentes de un municipio de Quindío, Colombia. *Rev Habanera Cienc Médicas*. 2020;19(5). Accessed July 25, 2022. http://scielo.sld.cu/scielo.php?script=sci_abstract&pid=S1729-519X2020000600016&lng=es&nrm=iso&tlng=es

[64] Ang YS, Frontero N, Belleau E, Pizzagalli DA (2020). Disentangling vulnerability, state and trait features of neurocognitive impairments in depression. Brain J Neurol.

[65] Cohen AJ, Dosa DM, Rudolph JL, Halladay CW, Heisler M, Thomas KS (2022). Risk factors for veteran food insecurity: findings from a National US Department of Veterans Affairs Food Insecurity Screener. Public Health Nutr.

[66] Brostow DP, Gunzburger E, Thomas KS (2017). Food Insecurity among Veterans: findings from the Health and Retirement Study. J Nutr Health Aging.

[67] Wang EA, McGinnis KA, Goulet J (2015). Food Insecurity and Health: data from the Veterans Aging Cohort Study. Public Health Rep.

[68] Pooler JA, Srinivasan M, Miller Z, Mian P (2021). Prevalence and risk factors for Food Insecurity among Low-Income US Military Veterans. Public Health Rep Wash DC 1974.

[69] Fang D, Thomsen MR, Nayga RM (2021). The association between food insecurity and mental health during the COVID-19 pandemic. BMC Public Health.

[70] Liebe RA, Adams LM, Hedrick VE (2022). Understanding the relationship between Food Security and Mental Health for Food-Insecure Mothers in Virginia. Nutrients.

[71] Zila-Velasque JP, Grados-Espinoza P, Quispe-Chura K, Valdiviezo-Morales CG, Diaz-Vélez C, Valladares-Garrido MJ (2022). Prevalence and factors associated with food insecurity in eight high-altitude cities in Peru during the second wave of the COVID-19 pandemic: a retrospective, cross-sectional study. BMC Public Health.

[72] Sabião TS, Mendonça RD, Meireles AL, Machado-Coelho GLL, Carraro JCC (2022). Food insecurity and symptoms of anxiety and depression disorder during the COVID- 19 pandemic: COVID-Inconfidentes, a population-based survey. SSM - Popul Health.

[73] Jiang Y, Jiang T, Xu LT, Ding L (2022). Relationship of depression and sleep quality, diseases and general characteristics. World J Psychiatry.

[74] Choi YH, Yang KI, Yun CH, Kim WJ, Heo K, Chu MK. Impact of Insomnia Symptoms on the Clinical Presentation of Depressive Symptoms: A Cross-Sectional Population Study. *Front Neurol*. 2021;12. Accessed July 25, 2022. https://www.frontiersin.org/articles/10.3389/fneur.2021.716097 10.3389/fneur.2021.716097 PMC8381020 34434165

[75] Ji X, Bastien CH, Ellis JG, Hale L, Grandner MA (2019). Disassembling insomnia symptoms and their associations with depressive symptoms in a community sample: the differential role of sleep symptoms, daytime symptoms, and perception symptoms of insomnia. Sleep Health J Natl Sleep Found.

[76] Plante DT (2021). The Evolving Nexus of Sleep and Depression. Am J Psychiatry.

[77] Valladares-Garrido MJ, Zapata-Castro LE, Domínguez-Troncos H (2022). Mental Health Disturbance after a major earthquake in Northern Peru: a Preliminary, cross-sectional study. Int J Environ Res Public Health.

[78] Bartone PT, Homish GG (2020). Influence of hardiness, avoidance coping, and combat exposure on depression in returning war veterans: a moderated-mediation study. J Affect Disord.

[79] To QG, Vandelanotte C, Cope K (2022). The association of resilience with depression, anxiety, stress and physical activity during the COVID-19 pandemic. BMC Public Health.

[80] Karaşar B, Canli D (2020). Psychological resilience and depression during the Covid-19 pandemic in Turkey. Psychiatr Danub.

[81] Haddadi P, Besharat MA (2010). Resilience, vulnerability and mental health. Procedia - Soc Behav Sci.

[82] Yuan K, Zheng YB, Wang YJ et al. A systematic review and meta-analysis on prevalence of and risk factors associated with depression, anxiety and insomnia in infectious diseases, including COVID-19: a call to action.*Mol Psychiatry*. Published online2022:1–9. doi:10.1038/s41380-022-01638-z 10.1038/s41380-022-01638-z PMC9168354 35668158

[83] Furer P, Walker JR, Chartier MJ, Stein MB (1997). Hypochondriacal concerns and somatization in panic disorder. Depress Anxiety.

[84] Warner V, Wickramaratne P, Weissman MM (2008). The role of fear and anxiety in the familial risk for major depression: a three-generation study. Psychol Med.

[85] Magnavita N, Soave PM, Antonelli M (2021). Prolonged stress causes Depression in Frontline Workers facing the COVID-19 Pandemic-A repeated cross-sectional study in a COVID-19 hub-hospital in Central Italy. Int J Environ Res Public Health.

[86] Bello UM, Kannan P, Chutiyami M (2022). Prevalence of anxiety and Depression among the General Population in Africa during the COVID-19 pandemic: a systematic review and Meta-analysis. Front Public Health.

[87] Cox RC, Olatunji BO (2016). A systematic review of sleep disturbance in anxiety and related disorders. J Anxiety Disord.

[88] Wang J, Zhou Y, Qian W, Zhou Y, Han R, Liu Z (2021). Maternal insomnia during the COVID-19 pandemic: associations with depression and anxiety. Soc Psychiatry Psychiatr Epidemiol.

[89] Ran L, Wang W, Ai M, Kong Y, Chen J, Kuang L (2020). Psychological resilience, depression, anxiety, and somatization symptoms in response to COVID-19: a study of the general population in China at the peak of its epidemic. Soc Sci Med 1982.

[90] Skalski SB, Konaszewski K, Büssing A, Surzykiewicz J (2021). Resilience and Mental Well-Being during the COVID-19 pandemic: serial mediation by Persistent thinking and anxiety about Coronavirus. Front Psychiatry.

[91] Ligeza N, Larson A, DeBeliso M, Resilience P, Stress P, Activity, BMI among United States Air National Guardsmen (2022). The COVID-19 pandemic. J Lifestyle Med.

[92] Kalin NH, Trauma, Resilience A, Disorders (2021). and PTSD Am J Psychiatry.

